# Protonation State of an Important Histidine from High Resolution Structures of Lytic Polysaccharide Monooxygenases

**DOI:** 10.3390/biom12020194

**Published:** 2022-01-24

**Authors:** Sanchari Banerjee, Sebastian J. Muderspach, Tobias Tandrup, Kristian Erik Høpfner Frandsen, Raushan K. Singh, Johan Ørskov Ipsen, Cristina Hernández-Rollán, Morten H. H. Nørholm, Morten J. Bjerrum, Katja Salomon Johansen, Leila Lo Leggio

**Affiliations:** 1 Department of Chemistry, University of Copenhagen, Universitetsparken 5, DK-2100 Copenhagen, Denmark; saba@chem.ku.dk (S.B.); sjm@chem.ku.dk (S.J.M.); tandrup@chem.ku.dk (T.T.); kehf@plen.ku.dk (K.E.H.F.); singhraushank@gmail.com (R.K.S.); mobj@chem.ku.dk (M.J.B.); 2 Department of Plant & Environmental Sciences, University of Copenhagen, Thorvaldsensvej 40, Frederiksberg C, DK-1871 Copenhagen, Denmark; jip@ign.ku.dk; 3 Department of Geoscience & Natural Resource Management, University of Copenhagen, Frederiksberg 5, DK-1958 Copenhagen, Denmark; ksj@ign.ku.dk; 4 The Novo Nordisk Foundation Center for Biosustainability, Technical University of Denmark, Kemitorvet Building 220, DK-2800 Kongens Lyngby, Denmark; crirol@biosustain.dtu.dk (C.H.-R.); morno@biosustain.dtu.dk (M.H.H.N.)

**Keywords:** polysaccharide monooxygenase, protonation, stereochemical properties, partial unrestrained refinement, auxiliary activities

## Abstract

Lytic Polysaccharide Monooxygenases (LPMOs) oxidatively cleave recalcitrant polysaccharides. The mechanism involves (i) reduction of the Cu, (ii) polysaccharide binding, (iii) binding of different oxygen species, and (iv) glycosidic bond cleavage. However, the complete mechanism is poorly understood and may vary across different families and even within the same family. Here, we have investigated the protonation state of a secondary co-ordination sphere histidine, conserved across AA9 family LPMOs that has previously been proposed to be a potential proton donor. Partial unrestrained refinement of newly obtained higher resolution data for two AA9 LPMOs and re-refinement of four additional data sets deposited in the PDB were carried out, where the His was refined without restraints, followed by measurements of the His ring geometrical parameters. This allowed reliable assignment of the protonation state, as also validated by following the same procedure for the His brace, for which the protonation state is predictable. The study shows that this histidine is generally singly protonated at the Nε2 atom, which is close to the oxygen species binding site. Our results indicate robustness of the method. In view of this and other emerging evidence, a role as proton donor during catalysis is unlikely for this His.

## 1. Introduction

Lytic Polysaccharide Monooxygenases (LPMOs) are a recently discovered group of copper metalloenzymes that depolymerize recalcitrant polysaccharides like lignocellulose and chitin by oxidative cleavage of the glycosidic bonds. LPMOs have been shown to boost the activities of other carbohydrate active enzymes, breaking down large crystalline/insoluble polysaccharides into glucans [1,2,3]. These sugars can then be fermented to bioethanol, a renewable form of energy [4]. This finding sparked the interest in LPMOs as it is a key step in boosting the production of biofuels from the plant biomass [5,6]. Extensive research is being carried out to understand the mechanism of the LPMO catalysis for effective industrial applications [7]. The presence of LPMOs or predicted LPMO-encoding genes has been established in many organisms, including microbes, fungi, algae, plants and insects [7]. Although their complete role in all the organisms is far from understood, some of the recent studies indicate biological roles of LPMOs in pathogenesis in plants [8,9] and human [10].

**Figure 1:**
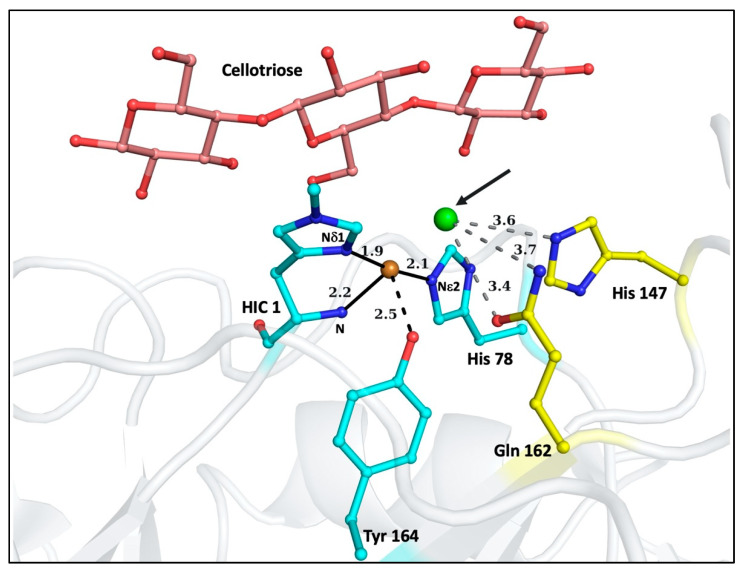
Structure of *Lentinus similis* AA9A (*Ls*AA9A), PDB ID: 5ACF showing its active site with the bound oligosaccharide cellotriose. The 1st coordination sphere of the copper (orange sphere) is formed by the residues His1, His78, and Tyr 164 (colored in cyan). The coordination distances are shown in Å. As the oligosaccharide is bound, the axial water is displaced in this structure, but is visible in Figure 4. At the equatorial position, chloride (green sphere) is bound instead of water. This position (pointed with a black arrow) is the presumed binding site for the activated oxygen species. The 2nd coordination sphere residues are His147 and Gln162 (colored in yellow). His147 is conserved across all AA9 LPMOs. The distances from His147 and Gln162 to the chloride in Å are shown in grey. The residues are represented in ball and stick representation. The figure was prepared in Pymol.

LPMOs are classified as Auxiliary Activities (AAs) in the Carbohydrate Active enZymes database, CAZy.org [11]. They have been extensively reviewed in [12,13,14,15,16]. There are eight families that are categorized as LPMOs: AA9, AA10, AA11, AA13, AA14, AA15, AA16, and AA17 (very recently discovered) [17]. There is a large diversity observed in the origin, substrate specificity, binding and cleavage reactions amongst the different LPMOs. Although the primary amino acid sequence of LPMOs is minimally conserved between families, the active site of the enzyme comprised of the Histidine brace (His-brace) is highly conserved across all LPMOs. The His-brace is made up of the Cu metal center coordinated with the imidazole ring Nδ1 and backbone N atoms of the terminal His and the imidazole ring nitrogen Nε2 atom of a His located later in the protein sequence. In AA9 and several other LPMOs, the Cu is also coordinated with a Tyr at an axial position and two water molecules: one axial (above the Tyr residue) and one equatorial (in the same plane as the two His residues) [18]. These residues constitute the primary co-ordination sphere of the enzyme. Figure 1 shows the active site of AA9A LPMO from *Lentinus similis* (*Ls*AA9A) bound to the oligosaccharide cellotriose. Due to the oligosaccharide binding, the axial water is displaced and a chloride ion binds from the crystallization condition at the position of the equatorial water. The active site is on a relatively flat surface site of the enzyme, which facilitates binding to large insoluble/crystalline substrates. This positioning eases the degradation process of the polysaccharides by the LPMOs as the large substrates do not need to enter binding pockets for enzyme activity. The structure of LPMOs from all families determined so far belong to the immunoglobulin G-like β-sandwich fold [19]. 

**Figure 2:**
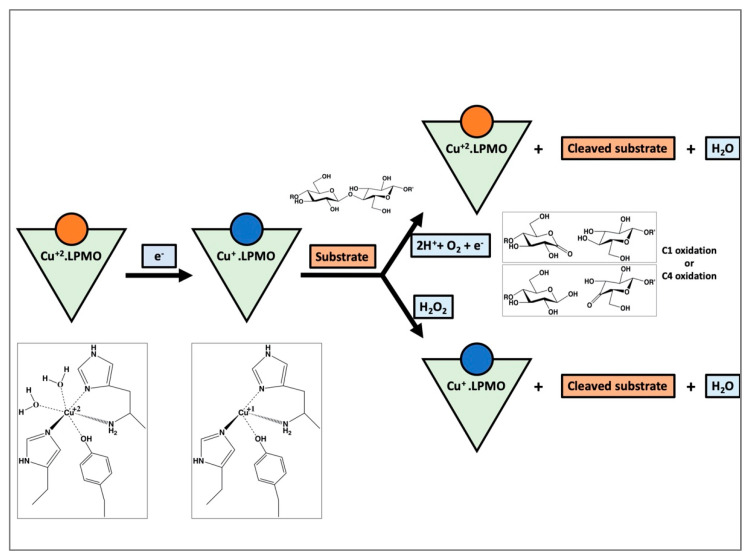
A simplified diagrammatic representation of the reaction mechanism of LPMOs exemplified by cellulose degradation. The reaction proceeds by reduction of the Cu from Cu(II) to Cu(I), substrate binding and the subsequent cleavage of the substrate by the oxygen species. Depending on the source of the oxygen species being either molecular oxygen or hydrogen peroxide, the reaction proceeds differently, yielding the cleaved substrate by C1 or C4 oxidation or both.

The general scheme of the activity of an LPMO is known; however, the full molecular mechanism is still elusive. A simplified diagrammatic representation of the putative mechanism for the oxidative cleavage of a substrate by an LPMO is shown in Figure 2. The reaction is initiated by the reduction of Cu(II) to Cu(I). Some of the electron donors aiding in reduction are (i) chemicals like ascorbic acid [16,20], (ii) the enzyme cellobiose dehydrogenase [21,22], and (iii) lignin from biomass [23]. A higher polysaccharide affinity for the reduced Cu(I) form of some LPMOs could suggest that reduction occurs before binding of the substrate [24]. After reduction and substrate binding, the oxygen species is bound at the equatorial position of the active site of the enzyme. The black arrow in Figure 1 (pointing to the chloride ion) denotes the position of the oxygen-species binding site. The oxygen species could be provided by either molecular O_2_ or H_2_O_2_, although the support for the latter is increasing [25,26,27,28]. After binding, the oxygen species is positioned appropriately near the substrate by the residues around the active site. This is likely followed by hydrogen abstraction from the substrate, hydroxylation and elimination of water, resulting in oxidation at either C1 or C4 atoms. However, recent detailed work on *Ls*AA9A shows that while glycosidic bond cleavage is dependent on H_2_O_2_, O_2_ is necessary for the production of oxidated oligosaccharides [28]. This suggests that rather than a strict preference for either co-substrates, at least some LPMOs follow more complicated reaction schemes where interactions between metal, oxygen species, protein and reductant determine the final outcome, which is highly sensitive to the reaction conditions.

The 1st and 2nd coordination spheres of transition metal complexes affect the structure and function of the metalloproteins [29]. While the residues of the 1st coordination sphere are mainly involved in bonding, the residues of the 2nd coordination sphere carry out their function via non-covalent interactions. Therefore, as in other transition metal complexes [29,30], the 2nd coordination sphere plays an important role in LPMO activity. 2nd sphere residues have been proposed to facilitate oxygen binding, proton transfer, and stabilization of intermediates through steric, electrostatic, and, especially, H-bonding interactions [27,29,30,31,32,33]. Due to very low sequence conservation across different LPMO families, there are different residues in the secondary coordination sphere that aid in the above reaction mechanism. In this study, the focus is on AA9 LPMOs, where a conserved His and Gln (His147 and Gln162 in *Ls*AA9A, Figure 1) form the 2nd coordination sphere and a strong H-bonding network has been shown in the crystal structures [34,35]. Of particular interest is the conserved His residue which has been shown by mutagenesis to be important for activity in an AA9 LPMO [31]. In addition to a role in positioning the oxygen species correctly along with the conserved Gln in AA9 LPMOs [27,31,32,33], a role has been discussed as a potential proton donor to the bound oxygen species [27,31,32,33] during the oxidation of the polysaccharides. 

Based on quantum mechanics/molecular mechanics (QM/MM calculations), Hedegård and Ryde investigated the effect of protonation state of His147 in *Ls*AA9A with a different co-substrate (O_2_ or H_2_O_2_) [27]. With O_2_, a double protonated His seems to be required as proton donor [27,31,32]. On the contrary, if the co-substrate is H_2_O_2_, the main role of His147 is the positioning of the co-substrate and not proton transfer, and a singly protonated His is sufficient [27]. Another study [33] shows that the process of O-O bond cleavage along with proton transfer from this double protonated His to oxygen is kinetically unfavorable [33]. Regardless which specific mechanism one wishes to investigate now or in the future, the protonation state of this secondary sphere residue is likely to affect the energy barriers obtained in QM/MM studies and is thus important to consider. 

Determining protonation states of residues in proteins experimentally is difficult. Nuclear Magnetic Resonance (NMR) spectroscopy titration and Neutron crystallography are the two most reliable methods [36,37,38,39,40]. While NMR is mostly amenable for small proteins, neutron crystallography is limited by low flux of neutron sources and size of the crystal, among others [41]. Several structures of LPMOs have been determined by NMR [42,43], but protonation states of key residues have not been specifically investigated with it. Neutron crystallography, on the contrary, has been used to investigate the protonation state of the His of the second co-ordination sphere in an AA9 from *Neurospora crassa*. Here the authors demonstrate that this His is singly protonated at Nε2 atom of the imidazole ring [32], but consider also the possibility that the active enzyme may be doubly protonated at low pH~5.0 and take part in proton transfer as the Nε2 atom is located near the O_2_ binding site.

In the present study we investigate the protonation state of this His residue using X-ray crystallographic data sets at resolutions better than 1.1 Å using partially unrestrained refinement as previously suggested [44]. Three structures of AA9 from *Ls*AA9A and *Thermoascus aurantiacus* (*Ta*AA9A) were determined in this study. Additionally we used the same protocol for re-refinement of four structures already deposited in the PDB. Although the secondary sphere His residue is not generally conserved in other families, AA10 LPMO from *Micromonospora aurantiaca* for which a high resolution X-ray crystal structure is available in the PDB [45], is an exception and thus is also included in the analysis. In order to validate the method, we have also used the same protocol to determine the (known) protonation state of the His in the His brace in these structures. The validation results show the robustness of our method for this study. Our results reveal that the conserved His residue of the 2nd coordination sphere in AA9 LPMOs is generally singly protonated. 

## 2. Materials and Methods

### 2.1. Crystallization

*Ls*AA9A was heterologously produced in *Escherichia coli* (*Ls*AA9A_*Ec*) using the LyGo platform [46]. The protein was purified using Q-Sepharose anion-exchange chromatography and Superdex 75pg size exclusion chromatography and finally concentrated to 13.8 mg/mL in the buffer: 20 mM Na-Acetate pH 5.5 and 150 mM NaCl. It was then incubated with Cu(II) acetate at equimolar ratio for 1 h before crystallization setup. The crystals for *Ls*AA9A_*Ec* were grown in a 24-well VDX plate by the hanging drop vapor diffusion method using 2 μL each of the protein and the crystallization condition, 0.1 M citric acid pH 3.5 and 3.0 M NaCl. The crystals were cryo-cooled in liquid nitrogen and data were collected at cryogenic temperature (100 K).

*Ta*AA9A was saturated with Cu(I) chloride under anaerobic environment. The filtered *Ta*AA9A preparation was then purified as described in [47]. The elution fractions corresponding to *Ta*AA9A were pooled and concentrated using an Amicon Ultra-15 centrifugal filter (3 kDa, Merck Millipore Ltd. Darmstadt, Germany). The concentrated *Ta*AA9A was again saturated with Cu(I) chloride under anaerobic environment and purified as described above. Finally, the Cu-loaded *Ta*AA9A was buffer exchanged in 20 mM MES pH 6.0 and concentrated to 26 mg/mL. The protein sample was then deglycosylated with ~0.05 units per mg *Ta*AA9A of endoglycosidase H (Roche Diagnostics A/S Hvidovre, Danmark). The final concentration of the protein was about 20.5 mg/mL in 20 mM MES pH 6.0, 125 mM NaCl. The protein sample was additionally incubated for 1 h with equimolar concentration of Cu(II) acetate prior to crystallization setup. The crystals of *Ta*AA9A were grown at room temperature using the sitting drop vapor diffusion method in MRC two-drop plates set up by an ORYX8 robot. These drops were composed of 0.3 μL of the protein sample *Ta*AA9A and 0.1 μL of the crystallization condition 0.1 M HEPES pH 7.5, 20 m M MgCl_2_ and 22 % (*w*/*v*) polyacrylic acid 5100 sodium salt. All the crystals were flash frozen in liquid nitrogen and data were collected at cryogenic temperature (100 K).

### 2.2. X-ray Data Collection and Structure Determination

High resolution X-ray diffraction data for *Ls*AA9A_Ec and *Ta*AA9A crystals were collected at the BioMAX beamline MAX IV laboratory, Lund. The two datasets for *Ls*AA9A_*Ec* were collected at a wavelength of 0.98 Å and 30% beam transmission for 360° with 0.1° oscillation. One dataset was collected for *Ta*AA9A at a wavelength of 0.95 Å and 40% beam transmission for 360° with 0.1° oscillation. 

All the three datasets were automatically processed through the EDNA pipeline [48] at the beamline, and were used for further structure determination (Table 1). Both the datasets for *Ls*AA9A_Ec extended to a resolution of 1.09 Å, while *Ta*AA9A data extended to a resolution of 1.06 Å.

The structure of *Ls*AA9A_*Ec* has been previously determined in the lab (PDB ID: 7PQR) in the space group P4_1_. The structure of *Ls*AA9A_*Ec*_1 is isomorphous to this initial structure. Therefore, its structure was determined by direct refinement of the starting model (7PQR) rigid body and restrained refinement in REFMAC5 [49] of the CCP4 suite [50] along with subsequent model building in COOT [51] and final anisotropic refinement. The structure of *Ls*AA9A_*Ec*_2 was instead determined by molecular replacement with the initial model using MOLREP [52] of the CCP4 suite, followed by rigid body and restrained refinement by REFMAC5 and manual model building by COOT. This difference arises because *Ls*AA9A_*Ec* crystallizes in the P4_1_ space group and can be indexed in 2 non-equivalent indexing with the same cell, and by chance, the EDNA processing pipeline chose the alternate indexing to the one used for the original structure. The dataset for *Ta*AA9A was isomorphous to the previously determined structure (PDB ID: 2YET) and its structure was determined by refinement in REFMAC5 and COOT with final anisotropic refinement. The quality of the structures were validated by checking the Ramachandran statistics using RAMPAGE [53] of CCP4 and through the PDB validation (Table 1). The coordinates for *Ls*AA9A_*Ec*_2 and *Ta*AA9A have been deposited in the PDB with accession codes 7PTZ and 7PU1, respectively. The figures of active sites were prepared in PyMOL 2.0.4 [54]. 

### 2.3. Re-Refinement of Other Structures from PDB

Four high resolution LPMO processed data sets and structures (PDB IDs: 5O2X, 4EIR, 5OPF, and 4QI8) were obtained from the PDB (Table 2). All of these structures have the conserved His in the 2nd co-ordination sphere and have resolutions higher than 1.1 Å [35,45,55,56].

### 2.4. Determination of His Protonation

**Figure 3:**
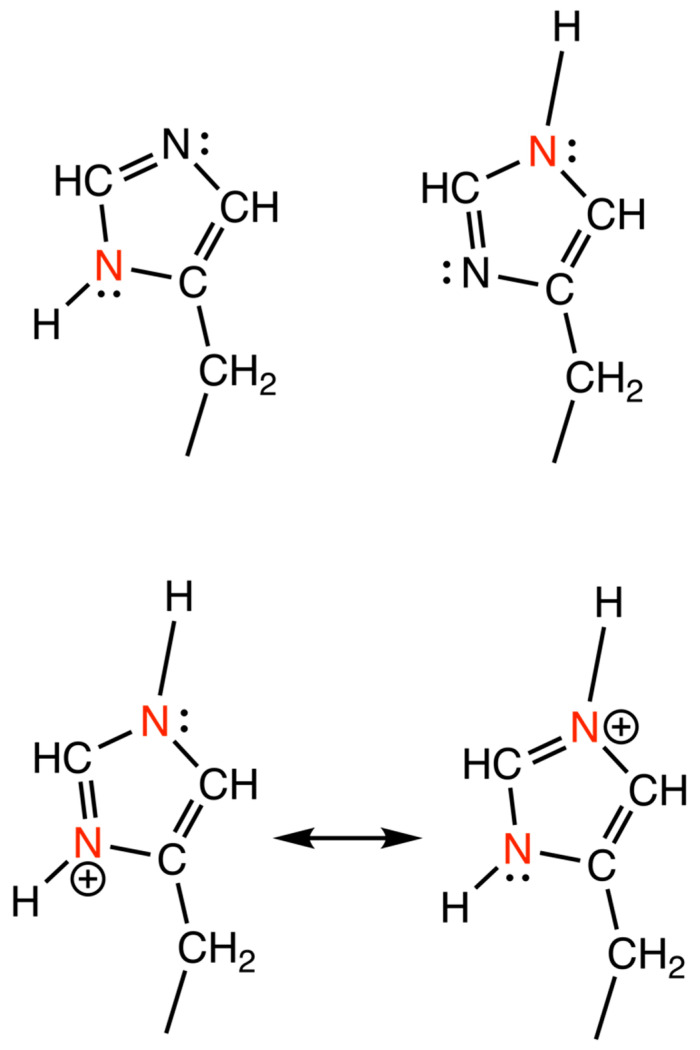
Protonated states of the imidazole ring of histidine side-chain. The top panel represents the singly protonated states with Nδ1 protonated in the left figure and Nε2 protonated in the right. The bottom panel represents the resonance forms of the doubly protonated state. All the protonated nitrogen atoms are colored in red. The figure was prepared using ChemDraw. The doubly deprotonated state is not physiologically relevant due to a p*K*_a_ of 14.5, hence not included in these structures.

Geometry of the His imidazole rings were studied as described by Malinska et al., 2015, where the four most sensitive stereochemical parameters in a His ring were used to derive two functions to determine the protonation states of the N atom in the imidazole ring of His [44]. These parameters are the bond lengths Nδ1-Cε1 (*X1* Å) and Cε1-Nε2 (*X2* Å) and the endocyclic angles -Nδ1- (*X3* degrees) and -Nε2- (*X4* degrees). The different protonation states are drawn in Figure 3.

The functions derived from these parameters are:

*his1* = −37.31*X1* + 15.57*X2* − 0.64*X3* + 0.76*X4* +17.30 (1)

*his2* = −2.16*X1* − 6.08*X2* + 0.56*X3* + 0.42*X4* − 94.46 (2)

If the equation *his1* is negative, only Nδ1 atom of the His ring is protonated. If the equation *his1* is positive, the value of *his2* is analyzed. If *his2* is negative, then, only Nε2 atom of the His ring is protonated. On the other hand, if *his2* is also positive, both the N atoms are assumed to be protonated. The doubly deprotonated state is excluded from our study as its p*K*_a_ is around 14.5 [57].

This study investigates the protonation state of the His in the 2nd co-ordination sphere. To prove the validity of our analysis, the two His residues of the His-brace have also been studied. His1 in the structures of *Ta*AA9A and PDBs 5O2X, and 4EIR are methylated as they are expressed in a filamentous fungal host. Therefore, they are excluded from our analysis. 

The pKa values of these histidine residues were calculated for comparison with the experimental results at the crystallization pH using PROPKA v 3.0.4 [58,59] and is listed in Table 3.

### 2.5. Refinement to Investigate Protonation

All the refinement programs used for protein structure determination including REFMAC5 of CCP4 have a built-in restraint target libraries. For His, these programs always consider it in a doubly protonated state during refinement. In order to investigate the protonation state of the nitrogen atoms in the His ring, it is important to remove the restraints for only the His residues studied in the structure. Thus in this work, restrained refinements were carried out on the structures using REFMAC scripts via command line in a Linux terminal, which allows greater control for restraining the residues. Two independent parallel refinements were carried out: (1) anisotropic restrained refinement for the whole structure, and (2) unrestrained refinement for the three His residues (two in cases where His1 is methylated) under investigation and anisotropic restrained refinement for the rest of the structure. While the 2nd type of refinement is the key protocol for determining the protonation state of His in this study, the former is carried out for comparisons of refinement statistics or changes in geometry. REFMAC scripts were customized to run these refinements. Due to the very high resolution of the structures, the R_work_ and R_free_ were well within acceptable limits. However, in order to ensure that the refinements have been carried out consistently, RMSD bond length of about 0.01–0.02 Å was used as a guiding reference by changing the variable, ‘weighing matrix’ to achieve this. In order to exclude restraints from a particular residue (His1 in the following case), the REFMAC script included the command: RESTRAINT EXCLUDE RESIDUE FROM 1 A TO 1 A ATOMS *. Evaluating the values of *his1* and *his2* functions for the structures where the His-only unrestrained refinement was carried out, will enable us to accurately determine the protonation states of these residues in the structure. NCS restraints were imposed for structures with two molecules in the asymmetric unit. The four geometric parameters of the imidazole ring of the histidines in the refined structures were measured in COOT.

In order to check that His-unrestrained refinement did not result in unreasonable geometry and/or unreasonable B factors average, atomic B factors for the His residues were compared to those of the whole structure in both restrained and His-only-unrestrained refinements using BAVERAGE of CCP4.

## 3. Results

Our present study aims to investigate the protonation state of the His at the 2nd co-ordination sphere from the AA9 LPMO structures. Here, the geometry of the His imidazole rings after partial unrestrained refinement were used to derive two functions (*his1/his2*) to determine the protonation states of the N atom. 

Six AA9A structures and one AA10 structure (containing the conserved His) with resolutions higher than 1.1 Å were used in this investigation. Three out of the six high resolution AA9A structures, two from *Lentinus similis* (two different data sets for the same enzyme) and one from *Thermoascus aurantiacus*, were determined in this study. Table 1 outlines the data collection and refinement statistics for these three structures. 

The data for all the three structures extends to very high resolutions. Although the completeness is low in the highest resolution shells, the CC_1/2_ suggests that inclusion of these data will positively contribute to refinement. In *Ls*AA9A_*Ec*_1, the completeness of the data cutting at resolutions of 1.09 Å and 1.16 Å are 84.6% and 99.8%, respectively. In *Ls*AA9A_*Ec*_2, the completeness of the data cutting at resolutions of 1.09 Å and 1.16 Å are 59.7% and 88.6%, respectively. In TaAA9A, the completeness of the data cutting at resolutions of 1.06 Å and 1.2 Å are 40.4% and 94.6%, respectively. Thus, all three datasets have good completeness beyond the threshold of 1.2 Å considered necessary for partial unrestrained refined, and therefore, we decided to extend the datasets to include higher resolutions, which can still contribute to improving the data/parameter ratio in refinement.

His147 in *Ls*AA9A and His164 in *Ta*AA9A were investigated for the protonation states at Nδ1 and Nε2 atoms of the imidazole ring of the histidines. The analysis was extended to the histidines at the active sites, where it is easy to conclude the protonation state of the imidazole ring, as one atom for each His was coordinated to the Cu ion. 

The comprehensive set of stereochemical restraint target values and their variances for proteins, EH99 [60] is used in all the refinement programs available in crystallographic structure determination suites. EH99 automatically assigns a double protonation state to histidine, whereby both the nitrogen atoms, Nδ1 and Nε2 have a covalently linked hydrogen atom. Therefore, to determine the true protonation state, it is essential to remove the geometric restraints from the histidine residues to be studied and carry out a specific type of refinement as described above in Section 2.5. Here, the His residues (of the primary and secondary coordination spheres) undergo unrestrained refinement, while the residues in the rest of the structure undergo restrained refinement. This His-only unrestrained refinement should allow these residues to assume the native geometry, if the data is of sufficient quality. The refinement statistics after His-only-unrestrained refinement are listed in Table 2. The values are very similar to completely restrained refinement statistics showing that un-restraining the histidines did not affect the geometry of the overall structure. 

The four geometrical parameters of the His ring were measured and the *his1* and *his2* functions calculated. These calculations are tabulated in Table 3.

For the His brace residues, one N-atom is coordinated to the Cu and cannot be protonated. The first His is excluded in those cases where it is methylated. If non-methylated, only its Nε2 atom can be protonated. For the secondary active site His, only Nδ1 can be protonated. To address the deviations from the expected results for the active site residues, the *his1* and *his2* values have been analyzed for the confidence level in predicting protonation. Furthermore, the average B-factors of the overall protein structure and the individual His residues from the parallel restrained and His-only unrestrained refinements have been compared in Table 4.

**Figure 4:**
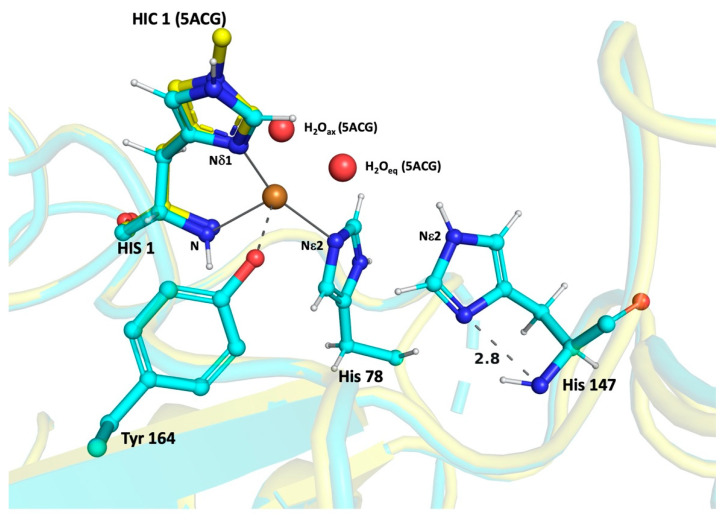
Results from His-only unrestrained refinement in *Ls*AA9A*Ec*_2 structure (cyan colored). His147 shows single protonation Nε2 atom. The Nis-brace residues (internal control) show their single protonation state. The H-bond distance between the Nδ1 and backbone N atoms of His147 is shown in Å. The structure of *Ls*AA9A_*Ao* (PDB id: 5ACG, yellow colored) is shown for comparison with 1^st^ His methylated (HIC) and the two water molecules bound to the Cu(II). The oxygen species binds at the position of H_2_O_eq_. The figure was prepared with Pymol.

The comparable values imply that removing the restraints from the histidines did not distort the geometry of the protein structure around these residues, thereby increasing the confidence level of our analysis. In all the cases, where there is an incorrect assignment of the protonated group for the His-brace residues, the values of *his1* and *his2* are found to be too low for correct estimation. Malinska and coworkers suggest that the protonation state for Nδ1 can be predicted with high probability when *his1* is less than −1.0. Similarly, the protonation state for Nε2 can be predicted with high probability if *his1* is positive and the *his2* is less than −1.0 [44]. Our results suggest that the His of the secondary co-ordination sphere is singly protonated at the Nε2 atom in most if not all structures. The results from our calculations and the predicted protonation sites are shown in Figure 4. For comparison, the structure of *Ls*AA9A_*Ao* expressed in *Aspergillus oryzae* (PDB id: 5ACG) was superposed on *Ls*AA9A_*Ec*_2 structure after its His-only unrestrained refinement, also showing the presence of the two water molecules.

## 4. Discussion

In the AA9 LPMO family, a conserved histidine in the secondary coordination sphere has been proposed, among other roles, to donate proton to the oxygen species via the Nε2 of its imidazole ring [31,32]. The present study was carried out to experimentally determine the protonation state of this conserved His residue using high resolution AA9 LPMO X-ray crystal structures, thereby adding experimental information to discussion of its role in proton transfer and a better starting basis for computational studies.

X-ray crystallography is a powerful tool for determining atomic resolution structures of proteins. X-rays can accurately determine the positions for almost all the atoms. Unfortunately, the hydrogen atom, being the lightest atom with one electron, has a very low scattering factor and the electron density is localized in the bond. Therefore, it is difficult to determine its position using this technique in the resolution ranges observed for proteins. However, it has been suggested that for structures with resolutions higher than 1.2 Å, the position of the hydrogen atoms can be predicted with a certain level of accuracy in protein structures [44]. We were able to carry out this study on seven different high resolution LPMO structures, which enabled us to validate our results across different enzymes within the AA9 family, as well as one AA10 enzyme. 

To evaluate the accuracy of the prediction of this analysis, protonation states for the active site His were also deduced. These histidines can only be singly protonated as one of the N atom (Nδ1 of His1 and Nε2 of the second His residue in the His-brace) is coordinated to Cu. There were four deviations from the expected protonation. These are His147 of *Ls*AA9A_*Ec*_1 (*his2* = 0.9156), His37 of the PDB structure 5OPF (*his1* = −0.5811), and His1 of both chains A (*his2* = 0.044) and B (*his1* = −0.1551) in the PDB structure 4QI8. The last three deviations are below the high confidence threshold, and with these exceptions the protonation states of all His-brace residues were accurately deduced from this study. This increases the confidence in our result on the secondary co-ordination sphere His which show that only Nε2 is protonated, while Nδ1 is in the deprotonated state. The only exception is *Ls*AA9A_*Ec*_1 structure, which shows protonation at both Nδ1 and Nε2 atoms, though with low confidence (as shown in Table 3). Examination of the micro-environment of this His residue in all the LPMO structures of this study show also that the distance of the Nδ1 atom from main chain N-H is between 2.75 to 2.95 Å. Therefore, this N is likely to serve as a H-bond acceptor and reinforcing the view that the His is unlikely to be doubly protonated.

From our study, where we observe only the single protonation of the conserved 2nd coordination sphere His at the Nε2 atom, we can conclude that this His will not be able to act as a proton donor to the oxygen species. Computational studies on *Ls*AA9A have also shown that the H_2_O_2_ co-substrate does not require proton transfer (and thus double protonation) [27,33]. A new experimental study further highlights that H_2_O_2_ is the co-substrate strictly required for *Ls*AA9A glycosidic bond cleavage activity [28]. Thus, at least for *Ls*AA9A there is currently no evidence of His147 double protonation or proton transfer being necessary for the mechanism. 

In neutron crystallography experiments on *Neurospora crassa* LPMO only Nε2 atom of this second co-ordination sphere His is found to be protonated [32], though it was suggested the residue might be deprotonated in the active form at lower pH. 

Table 3 shows the pH of the crystallization condition for all the structures used in this study, which range from 3.5–7.5. Though there might be deviation from the nominal crystallization pH, the pH range suggests that in spite of the crystallization conditions being acidic for some cases, it does not change the protonation state of the LPMO. Based on our PROPKA calculations in Table 3, the p*Ka* of the 2nd coordination sphere His is in the range of 4.64–5.07, which shows that it will be neutral at the pH of 6.0–7.0, where LPMO activity is often measured. This also implies that for the structures in this study, where crystallization pH is very low, we would expect some doubly protonated state, which might be what the low confidence doubly protonated state of the *Ls*AA9A_*Ec*_1 structure shows. As all other structures though generally show single protonation, it is likely that the *p*Kas of this His might be even lower than those predicted by PROPKA. 

Taken together, our study using partial unrestrained refinement of high-resolution LPMO structures shows that the secondary co-ordination sphere His is primarily singly protonated at Nε2 atom at all crystallization conditions and pH, arguing against a general role as proton donor, as the most recent computational studies also suggest. However, the possibility that the presence of the oxygen species/saccharide substrate brings changes in the His *p*k_a_ directly or through structural changes cannot be completely excluded, and neither is the possibility that this doubly protonated species is transient and thus not possible to characterize by X-ray crystallography. 

In conclusion, the scope of this study was to probe into the protonation state of a specific His residue. Based on our internal validation using the His-brace, the *his1*/*his2* assignments are highly reliable (except those in low confidence zone). More and more X-ray structures of enzymes are being determined to better than 1.2 Å resolution, and the role of His in catalysis is widespread in many types of enzymes. Thus, based on the current experience with LPMOs, we recommend the use of the partial restrained protocol devised by [44] as a complement to neutron crystallography in order to determine experimentally the protonation state of important histidines.

## Figures and Tables

**Table 1:** Data Collection and Refinement Statistics for the three high resolution structures determined.

	*Ls*AA9A_*Ec*_1	*Ls*AA9A_*Ec*_2	*Ta*AA9A
Beamline	BioMAX, MAXIV, Lund	BioMAX, MAXIV, Lund	BioMAX, MAXIV, Lund
Wavelength [Å]	0.9762	0.9762	0.9538 Å
Space group	*P*4_1_	*P*4_1_	*P*2_1_
No. of mols/asymmetric unit	1	1	2
Cell parameters			
(a, b, c) [Å]	48.47, 48.47, 109.72	48.66, 48.66, 109.59	37.65, 89.05, 70.47
(α,β,γ) [º]	90.0	90.0	90.0, 103.3, 90.0
Resolution [Å]	48.5–1.09 (1.15–1.09) *	48.7–1.09 (1.16–1.09)	68.6–1.06 (1.12–1.06)
Completeness [%]	97.2 (84.6)	91.3 (59.7)	84.2 (40.4)
R_meas_ [%]	5.3 (36.1)	5.9 (113.4)	5.4 (43.4)
I/σ(I)	7.21 (3.37)	19.3 (1.5)	18.6 (3.6)
CC_1/2_ [%]	99.8 (88.6)	99.9 (73.1)	99.9 (89.4)
Unique reflections	102,068 (15,109)	96,541 (10,733)	171,807 (12,525)
Observed reflections	1,163,180 (71,657)	1,157,797 (63,079)	1,126,749 (54,423)
Redundancy	11.4 (4.7)	12.0 (5.9)	6.6 (4.3)
No. mol./ASU	1	1	2
R_Work_ [%]	11.23	12.35	11.73
R_Free_ [%]	12.75	13.93	14.00
RMSD			
Bond lengths [Å]	0.0191	0.0196	0.0193
Bond Angles [°]	2.1238	2.0322	2.3041
Ramachandran Statistics (%)			
Favored	94.8	94.8	98.4
Allowed	5.2	5.2	1.1
Outlier	0.0	0.0	0.4

* Highest resolution shell shown in parenthesis.

**Table 2:** Refinement statistics after restrained and His-unrestrained refinement of the seven structures in this study.

	Crystallization Condition	No. of Mols/Asymmetric Unit	Resolution (Å)	Restrained Refinement	Histidine-Unrestrained Refinement		
				Final R_Work_/R_Free_ [%]	Final R_Work_/R_Free_ [%]	Final RMSD Bond lengths [Å]	Final RMSD Bond Angles [°]
*Ls*AA9A_*Ec*_1	0.1 M citric acid pH 3.5 and 3.0 M NaCl	1	1.09	11.04/12.36	11.39/12.82	0.0131	1.8818
*Ls*AA9A_*Ec*_2	0.1 M citric acid pH 3.5 and 3.0 M NaCl	1	1.09	12.16/13.79	12.62/13.93	0.0170	1.9397
*Ta*AA9A	0.2 M MgCl_2_, 0.1 M HEPES pH 7.5, 22% polyacrylic acid	2	1.06	11.94/14.17	11.80/12.70	0.0121	2.0481
5O2X	1.6 M ammonium sulfate, 0.1 M citric acid 4.0	1	0.95	11.29/12.53	11.39/12.58	0.0139	1.9586
4EIR	20% PEG 3350, pH 6.7	2	1.10	12.07/13.52	11.20/12.73	0.0147	1.8145
5OPF	0.04 M potassium phosphate monobasic, 16% *w*/*v* PEG8000, 20% *v*/*v* glycerol	1	1.08	11.49/13.86	11.79/14.02	0.0124	1.7270
4QI8	0.2M ammonium nitrate, 20% (*w*/*v*) PEG 3350, pH 7.0	2	1.10	11.98/14.60	12.08/14.62	0.0161	1.8468

**Table 3:** Geometrical parameters and estimation of protonation state after His-only unrestrained refinement. The PROPKA predicted p*Ka* values for these histidine residues are also given in the last column. These p*Ka* values denotes the transition of the His imidazole ring from doubly protonated, positively charged state to singly protonated neutral form. The grey boxes represent values of *his1* or *his2* between +1 and −1, and thus with low confidence in predicting the protonation states. For each structure, the first two (or one in some cases) represent the active site His residues and the last residue represents the 2nd coordination sphere His.

	Nδ1-Cε1 (*X*_1_) (Å)	Cε1-Nε2 (*X*_2_) (Å)	-Nδ1- (*X*_3_) (deg)	-Nε2- (*X*_4_) deg)	*his1*	*his2*	Protonated Group	Predicted p*Ka*
*Ls*AA9A_*Ec*_1								
1	1.37	1.33	105.04	108.92	2.3922	−0.9368	Nε2	5.65
78	1.34	1.31	107.76	106.57	−0.3255	−0.2142	Nδ1	3.70
147	1.35	1.37	107.88	110.02	2.7804	0.9156	Nδ1 + Nε2	5.07
*Ls*AA9A_*Ec*_2								
1	1.36	1.33	105.33	107.53	1.5237	−1.3366	Nε2	5.53
78	1.33	1.37	110.10	106.26	−0.751	0.6228	Nδ1	3.49
147	1.34	1.43	107.53	107.20	2.1689	−0.808	Nε2	4.95
*Ta*AA9A_A chain								
86	1.39	1.33	105.37	105.03	−1.5224	−2.429	Nδ1	2.78
164	1.32	1.33	105.30	109.14	4.2605	−0.5908	Nε2	4.80
*Ta*AA9A_B chain								
86	1.40	1.31	104.16	106.83	−0.0649	−2.2506	Nδ1	2.83
164	1.31	1.33	106.09	107.37	2.7832	−0.8702	Nε2	4.82
5O2X								
86	1.36	1.30	105.81	103.08	−2.6326	−2.7544	Nδ1	2.45
163	1.34	1.34	105.72	107.36	2.0476	−1.2072	Nε2	4.63
4EIR_A chain								
84	1.35	1.30	107.34	106.85	−0.3731	−0.2926	Nδ1	2.21
157	1.30	1.32	106.39	108.31	3.5234	−0.225	Nε2	4.68
4EIR_B chain								
84	1.33	1.32	108.83	105.27	−1.4691	−0.2002	Nδ1	2.24
157	1.31	1.33	106.17	107.73	3.0056	−0.6742	Nε2	4.67
5OPF								
37	1.33	1.32	105.02	103.23	−0.5811	−3.1906	Nδ1	3.44
144	1.32	1.30	106.03	103.60	−0.8842	−2.3264	Nδ1	2.18
216	1.30	1.34	103.87	104.49	2.5444	−3.3622	Nε2	4.83
4QI8_A chain								
1	1.36	1.28	105.25	110.2	2.8256	0.044	Nδ1 + Nε2	4.29
72	1.34	1.30	107.14	104.41	−1.7260	−1.4078	Nδ1	2.19
146	1.33	1.36	105.18	105.13	1.3833	−2.5462	Nε2	4.75
4QI8_B chain								
1	1.37	1.32	106.58	107.07	−0.1551	−0.7906	Nδ1	4.23
72	1.36	1.32	107.96	105.84	−1.5996	−0.5128	Nδ1	2.11
146	1.32	1.32	105.02	106.52	2.2928	−1.7872	Nε2	4.64

**Table 4:** Average B factors for overall protein structure and the His residues after restrained and His-only restrained refinement.

Histidine Residue Number in This Study	B_average_ (Å^2^) from Restrained Refinement	B_average_ (Å^2^) from His-Only Unrestrained Refinement
*Ls*AA9A_*Ec*_1 Overall_protein-chain	14.1	14.1
1	12.7	12.8
78	13.1	13.1
147	14.1	14.2
*Ls*AA9A_*Ec*_2 Overall_protein-chain	17.5	17.7
1	15.7	15.8
78	15.8	15.9
147	17.5	17.7
*Ta*AA9A Overall_protein-chainA	12.5	12.3
86_chainA	9.4	9.4
164_chainA	10.0	10.0
Overall_protein-chainB	12.9	12.8
86_chainB	9.8	9.7
164_chainB	10.7	10.7
5O2X Overall_protein	7.1	7.0
86	5.8	5.7
163	6.8	6.8
4EIR Overall_protein-chainA	17.8	16.9
84_chainA	12.7	12.6
157_chainA	14.1	14.0
Overall_protein-chainB	13.1	12.8
84_chainB	10.8	10.7
157_chainB	11.4	11.4
5OPF Overall_protein	9.4	9.3
37	8.0	8.0
144	7.2	7.1
216	7.8	7.8
4QI8 Overall_protein-chainA	17.8	16.9
1_chainA	10.7	10.6
72_chainA	27.1	26.5
146_chainA	13.5	13.4
Overall_protein-chainB	13.0	12.8
1_chainB	10.2	10.2
72_chainB	28.4	27.3
146_chainB	12.4	12.3

## Data Availability

Publicly available datasets were analyzed in this study. These data can be found here: https://www.rcsb.org, 17 January 2022. New PDB datasets from this study are 7PTZ and 7PU1.
